# What anthropometric indicators are associated with insulin resistance? Cross-sectional study on children and adolescents with diagnosed human immunodeficiency virus

**DOI:** 10.1590/1516-3180.2021.0303.27052021

**Published:** 2022-01-05

**Authors:** Carlos Alencar Souza Alves, Priscila Custódio Martins, Luiz Rodrigo Augustemak de Lima, Diego Augusto Santos Silva

**Affiliations:** I MSc. Doctoral Student, Postgraduate Program on Physical Education, Universidade Federal de Santa Catarina (UFSC), Florianópolis (SC), Brazil.; II MSc. Doctoral Student, Postgraduate Program in Physical Education, Universidade Federal de Santa Catarina (UFSC), Florianópolis (SC), Brazil.; III PhD. Adjunct Professor, Institute of Physical Education and Sport, Universidade Federal de Alagoas (UFAL), Maceió (AL), Brazil.; IV PhD. Associate Professor, Department of Physical Education, Universidade Federal de Santa Catarina (UFSC), Florianópolis (SC), Brazil.

**Keywords:** Anthropometry, Child, Adolescent, HIV, Body composition, Skinfold thickness, Children, Body fat, Youths, Human immunodeficiency virus

## Abstract

**BACKGROUND:**

Studies that test associations between anthropometric indicators and insulin resistance (IR) need to provide better evidence in the context of the pediatric population (children and adolescents) with human immunodeficiency virus (HIV), as anthropometric indicators present a better explanation of the distribution of body fat.

**OBJECTIVE:**

To test the associations between anthropometric indicators and insulin resistance (IR) among children and adolescents diagnosed with HIV.

**DESIGN AND SETTING:**

Cross-sectional study on 65 children and adolescents (8-15 years) infected with HIV through vertical transmission conducted at the Joana de Gusmão Children's Hospital, Florianópolis, Brazil.

**METHODS:**

The anthropometric indicators measured were the abdominal (ASF), triceps (TSF), subscapular (SSF) and calf (CSF) skinfolds. The relaxed arm (RAC), waist (WC) and neck (NC) circumferences were also measured. Body mass index (BMI) was calculated from the relationship between body mass and height. IR was calculated through the Homeostasis Model Assessment for Insulin Resistance (HOMA-IR). Simple and multiple linear regression analyses were used.

**RESULTS:**

After adjustment for covariates (sex, bone age, CD4+ T lymphocytes, CD8+ T lymphocytes, viral load, and physical activity), associations between IR and models with SSF and CSF remained. Each of these explained 20% of IR variability. For females, in the adjusted analyses, direct associations between IR and models with ASF (R² = 0.26) and TSF (R² = 0.31) were observed.

**CONCLUSIONS:**

SSF and CSF in males and ASF and TSF in females were associated with IR in HIV-infected children and adolescents.

## INTRODUCTION

Antiretroviral treatment (ART) and human immunodeficiency virus (HIV) infection itself can cause side effects in individuals with HIV.^1^ Among the adverse effects, visible changes in the body such as lipodystrophy syndrome^2^ and metabolic changes such as dyslipidemia and insulin resistance (IR)^3^ are among the most common adverse effects. IR is defined as lower capacity of insulin to instigate use of glucose by adipose tissue and muscles, or which leads to expansion of pancreatic insulin formation.^4^

HIV and continued use of ART are considered to be facilitators for development of IR in the pediatric population.^4^ HIV infection, opportunistic infections and intestinal inflammation can culminate in changes to inflammatory cytokines, such as soluble tumor necrosis factor and hormones such as adiponectin and reduced leptin, which impairs glucose homeostasis.^5,6^ In addition, changes to CD4+ and CD8+ T-cell functions may impair glycolysis, which may adversely influence glucose metabolism.^6^ Specifically, ART protease inhibitors have been associated with hyperglycemia and glucose tolerance in adults diagnosed with HIV^7^ and may inhibit the action of glucose transporter (GLUT4), thus resulting in decreased insulin-mediated glucose intake by muscle and adipose tissue.^1^ In addition, changes to the body fat distribution pattern may result in changes to the hormonal secretory system of adipose tissue and generate a chronic inflammatory profile, which facilitates IR development.^8^

Overweight is among the factors that contribute to the onset of IR in young people without HIV,^8^ especially increased body fat. To assess body fat, anthropometric indicators are commonly used.^9^ Different measurements have been directly associated with IR, such as neck circumference,^10^ waist circumference (WC) and body mass index (BMI) in different populations.^11^ In pediatric populations diagnosed with HIV, it has been reported that WC and BMI were directly associated with development of IR.^12,13^ This underscores the relevance of easily and cheaply obtained anthropometric indicators for assessing associations with IR in HIV-infected children and adolescents.

Although a relationship between anthropometric indicators (WC and BMI) and IR in HIV-infected children and adolescents has been identified, the indicators used have not enabled analysis of fat distribution.^12,14,15^ Skinfolds, which measure the thickness of the underlying layer of subcutaneous fat, are anthropometric indicators that have a direct association with reference methods for body fat assessment among young people diagnosed with HIV.^16^ Furthermore, skinfolds are anthropometric markers that indicate accumulation of body fat in the peripheral region (i.e. triceps skinfold, TSF) and central region (i.e., subscapular skinfold, SSF; and abdominal skinfold, ASF).

Studies that test associations between skinfolds and IR need to provide better evidence in the context of the pediatric population (children and adolescents) with HIV, as anthropometric indicators present a better explanation of the distribution of body fat.

## OBJECTIVE

The purpose of this study was to test associations between anthropometric indicators and IR in a pediatric population (eight to 15 years of age) with HIV.

## METHODS

### Population and sample

This was a cross-sectional study, conducted from 2015 to 2016 (November to June) in a city in southern Brazil. The research protocol was approval by the human research ethics committee of Universidade Federal de Santa Catarina (UFSC) (protocol number: 49691815.0.0000.0121; date of approval: February 15, 2016) and was also approved by the research ethics committee of Hospital Infantil Joana de Gusmão, Florianópolis, Santa Catarina (protocol number: 037/2015; date of approval: October 20, 2015).

### Participants

Children and adolescents aged between eight and 15 years, with vertical transmission of HIV, were recruited for the study and were followed up clinically at the Hospital Infantil Joana de Gusmão, Florianópolis, Brazil. Eighty-three eligible patients were found. Three patients were excluded from the sample because they presented severe encephalopathy and because they were unable to walk. Three were excluded because we were unable to contact them, four because they had been transferred to another hospital and four because they refused to participate in the research; and another four were losses during the data collection. The final sample consisted of 65 subjects.

The inclusion criteria were the following: a) presence of information in the medical record to prove that HIV infection had been transmitted from mother to child; b) age between 8 and 15 years; c) ability to stand and communicate; and d) presence of laboratory and clinical information about the infection. The exclusion criteria were the following: a) presentation of contraindication against vigorous intensity exercises and existence of motor disability; b) problems that made speech, hearing and/or cognition impossible; c) presence of diseases that change body composition, except for HIV infection itself; and d) use of immunotherapies and regular use of diuretics. Individuals with any pathological condition other than HIV were excluded from the study.

The sample size was calculated *a posteriori*, taking into account the type I error (α = 0.05) and type II error (β = 0.80) for testing associations between anthropometric indicators and IR, with an average effect size (0.50).^17^ For simple and multiple linear regression analyses, the posterior analysis indicated that with α = 0.05 and β = 0.80, a sample of 65 HIV+ children and adolescents would make it possible to find associations between anthropometric indicators and IR, with an effect size of 0.50.^17^ All calculations were performed using the G* Power software version 3.1.9.2 (Universität Düsseldorf, Germany).

### Dependent variable

To check IR, we used the Homeostasis Evaluation Model for Insulin Resistance Index (HOMA-IR), calculated through the mathematical model described by Matthews et al.^18^ We applied the following equation: HOMA-IR = fasting blood glucose (mg/dl) x insulin (μIU/ml). In the mornings, fasting blood samples (15 ml) were collected to measure glucose and insulin concentrations. Glucose levels were determined using the oxidase method (Wiener CB 400i; Wiener Lab Group, Rosario, Argentina) and insulin levels were measured using the chemiluminescence method (Roche Diagnostics Elecsys, Indianapolis, United States).

The gold standard for IR evaluation is the hyperinsulinemic-euglycemic clamp.^4^ However, this technique is expensive and invasive in the research context, and use of alternative methods for IR identification, such as HOMA-IR and the insulin sensitivity check index (QUICKI), is more frequent.^4,19^ HOMA-IR has high correlation (r = 0.88) with the hyperinsulinemic-euglycemic clamp in the pediatric population and demonstrates two evaluation parameters: plasma insulin and glucose.^19^

### Independent variables

The anthropometric indicators measured were skinfolds: ASF, TSF, SSF and CSF; relaxed arm circumference (RAC); waist circumference (WC) and neck circumference (NC). Body mass index (BMI) was calculated from the relationship between body mass and square of height.

Standardization of measurements was performed in accordance with the guidelines of the International Society for the Advancement of Kinanthropometry (ISAK) by an ISAK level 1 certified anthropometrist. A sample of 32 children of the same age group was also measured to calculate the technical error of intra-rater measurement (TEM).^20^

To measure the skinfold thickness, a caliper plicometer (Cescorf, Porto Alegre, Brazil) with a resolution of 0.1 mm was used. Anthropometric tape without elasticity was used to measure body circumferences (Sanny, São Paulo, Brazil) with a unit of measurement of 0.1 cm. Portable digital scales (Tanita, 180 Tokyo, Japan) were used to measure body mass, with a total capacity of up to 150 kg, and with a resolution of 0.1 kg. A stadiometer (AlturaExata, Belo Horizonte, Brazil) was used for height verification, with a measuring capacity from 115 cm to 210 cm and a unit of measurement of 0.1 cm.

### Control variables

Information on viral load, CD4+ T lymphocyte count (%) and CD8+ T lymphocyte count (%) was obtained from each participant's medical records. Bone age was assessed by means of wrist-carpal radiography, in the radiology sector of Joana de Gusmão Children's Hospital (JGCH). For this measurement, international standardization was used.^21^ Bone age was treated as a continuous variable.

The GT3X-Plus Actigraph accelerometer (Manufacturing Technology Inc., Fort Walton Beach, United States) was used to measure moderate to vigorous-intensity physical activity (MVIPA), continuously over a seven to 14-day period that included weekend days. To ensure data reliability, the participants were asked to always use the accelerometer on the right side, located at the waist, throughout the day, and only to remove it for activities such as bathing, water activities and sleep. For data analysis, we considered records that extended across at least four days (three on weekdays and one on weekends) for a period of 10 hours or more, after removing times of non-use consisting of at least 60 one-minute records of successive zeros. The cutoff points proposed by Evenson^22^ were used to quantify the MVIPA minutes, and these were adjusted according to the proportional time for which the youths remained awake (14 hours). Verbal and written instructions were made available to participants and guardians before the device was used.

### Statistical treatment

Firstly, descriptive analyses were performed on the data (median and interquartile range). Kurtosis and asymmetry analyses were then used to verify data normality (range from −2 to + 2),^23^ in addition to histogram analysis to identify data distribution normality. Student's t-test and the chi-square test were used to identify sex (male/female) differences. Simple and multiple linear regression were used to test associations between outcomes and exposures, respectively. For the multiple linear regressions, control variables (sex, bone age, CD4+ T lymphocytes, CD8+ T lymphocytes, viral load and physical activity) were used. Regression coefficients (β), 95% confidence intervals and determination coefficients (R²) for each model analyzed and diagnoses of multicollinearity (VIF) were estimated. For descriptive analyses and simple and multiple linear regressions, the Statistical Package for the Social Sciences software (IBM SPSS Statistics, Chicago, United States), version 22.0, was used, with P ≤ 0.05. All analyses were performed stratified according to sex (male/female), which can be justified through existence of sexual dimorphism, because as age increases, secretion of sex hormones can interfere with the amount of body fat.^24^

## RESULTS

Sixty-five children and adolescents aged 8-15 years (30 males and 35 females), diagnosed with HIV, participated in the study. There were differences between the sexes, such that the females had higher SSF (P < 0.001) and CSF (P = 0.050) than the males. Regarding physical activity, the male adolescents did more minutes/day than the females (P = 0.022) (Table 1).

**Table 1:** Characteristics of children and adolescents diagnosed with human immunodeficiency virus (HIV), stratified according to sex (n = 65)

		Male (n = 30)		Female (n = 35)		
		Mean (SD)	Min; Max	Mean (SD)	Min; Max	P-value
Chronological age (years)		12.24 (2.19)	8.36; 14.79	12.16 (2.10)	8.01; 15.01	0.401
Bone age (years)		11.66 (2.83)	6.00; 17.00	12.31 (2.55)	5.75; 16.00	0.397
Height (cm)		147.72 (13.78)	116.80; 173.20	147.00 (12.63)	114.20; 167.00	0.694
Body mass (kg)		39.45 (12.24)	21.90; 72.40	40.38 (10.94)	18.70; 65.10	0.765
ASF (mm)		8.87 (4.07)	4.00; 18.00	10.74 (3.76)	5.00; 24.00	0.492
SSF (mm)		6.24 (2.00)	4.00; 13.00	8.23 (3.99)	4.00; 19.00	< **0.001**
TSF (mm)		8.33 (2.38)	5.00; 13.00	10.74 (3.76)	6.00; 20.00	0.136
CSF (mm)		9.33 (2.84)	3.00; 16.00	11.06 (3.87)	6.00; 20.00	**0.050**
WC (cm)		63.59 (6.43)	53.00; 75.00	62.80 (6.72)	51.00; 82.00	0.870
RAC (cm)		20.97 (3.15)	16.00; 29.00	21.54 (3.03)	16.00; 27.00	0.880
NC (cm)		29.53 (3.41)	24.00; 38.00	28.34 (1.99)	23.00; 32.00	< **0.001**
BMI (kg/cm^2^)		17.69 (2.69)	12.62; 24.19	18.40 (2.74)	14.38; 24.80	0.671
Moderate-vigorous physical activity (minutes/day)		58.32 (31.90)	12.50; 141.80	39.51 (18.17)	10.50; 73.90	**0.022**
Viral load (log)		2.22 (1.03)	1.60; 5.04	2.11 (0.93)	1.60; 4.97	0.644
CD4+ T lymphocytes (cells/mm^3^)		861.50 (364.55)	196.00; 1.811	854.31 (375.71)	135.00; 1731.00	0.552
CD8+ T lymphocytes (cells/mm^3^)		44.27 (13.08)	405.00; 3.583	125.36 (508.49)	495.00 2.297	0.079
HOMA-IR		1.27 (0.83)	0.37; 3.79	1.56 (1.09)	0.27; 6.01	0.338
		**n**	**(%)**	**n**	**(%)**	
**Current ART usage**						0.846
	Yes; with protease inhibitor	19	48.70	20	51.30	
	Yes; without protease inhibitor	6	40.00	9	60.00	
	Not used	5	45.50	6	54.50	
**Sexual maturation**						0.672
	Prepubertal	8	53.30	7	46.70	
	Pubertal	21	44.70	26	55.30	
	Postpubertal	0	66.70	1	33.30	

ASF = abdominal skinfold; SSF = subscapular skinfold; TSF = triceps skinfold; CSF = calf skinfold; BMI = body mass index; RAC = relaxed arm circumference; WC = waist circumference; NC = neck circumference; IR = insulin resistance; cm = centimeters; mm = millimeters; SD = standard deviation; Min = minimum; Max = maximum; ART = antiretroviral drugs; HOMA-IR = Homeostasis Model Assessment for Insulin Resistance.

Among the males, direct associations were observed in simple linear regressions between IR and SSF (R² = 0.24), CSF (R² = 0.15), WC (R² = 0.15), RAC (R² = 0.11), NC (R² = 0.12) and BMI (R² = 0.15). After adjustment for covariates (sex, bone age, CD4+ T lymphocytes, CD8+ T lymphocytes, viral load and physical activity), associations between IR and models with SSF and CSF remained, and each of these explained 20% of the IR variability (Table 2).

**Table 2:** Simple and multiple linear regressions between insulin resistance and anthropometric indicators among male and female children and adolescents diagnosed with HIV (n = 65)

Male (n = 30)									
	Simple				Multiple*				
	β (95% CI)	SE	R^2^	P	β (95% CI)	SE	R^2^	P	VIF
ASF (mm)	0.06 (−0.02; 0.13)	0.04	0.42	0.12	0.05 (−0.04; 0.14)	0.04	0.08	0.28	1.30
SSF (mm)	0.21 (0.07; 0.35)	0.07	0.24	**< 0.01**	0.17 (0.01; 0.33)	0.07	0.20	**0.04**	1.34
TSF (mm)	0.09 (−0.03; 0.21)	0.06	0.03	0.16	0.05 (−0.09; 0.19)	0.07	0.05	0.47	1.22
CSF (mm)	0.12 (0.02; 0.23)	0.05	0.15	**0.02**	0.14 (0.01; 0.28)	0.06	0.20	**0.04**	1.34
WC (cm)	0.05 (0.01; 0.10)	0.02	0.16	**0.02**	0.03 (−0.04; 0.11)	0.03	0.07	0.33	2.64
RAC	0.09 (0.01; 0. 19)	0.05	0.11	**0.04**	−0.02 (−0.21; 0-16)	−0.08	0.03	0.82	3.54
NC	0.09 (0.01; 0.18)	0.04	0.12	**0.03**	0.02 (−0.13; −0.18)	0.07	0.04	0.72	3.16
BMI	0.13 (0.02; 0.24)	0.05	0.15	**0.01**	0.07 (−0.13; 0.28)	0.10	0.06	0.45	3.17
**Female (n = 35)**									
ASF (mm)	0.10 (−0.57; 1.22)	0.44	0.20	< 0.01	0.11 (0.02; 0.20)	0.04	0.26	**0.01**	1.57
SSF (mm)	0.08 (−0.01; 0.17)	0.05	0.05	0.09	0.06 (−0.04; 0.17)	0.05	0.12	0.23	1.41
TSF (mm)	0.13 (0.04; 0.22)	0.04	0.18	**< 0.01**	0.14 (0.05; 0.24)	0.04	0.31	**< 0.01**	1.32
CSF (mm)	0.08 (−0.01; 0.18)	0.05	0.05	0.09	0.10 (−0.01; 0.22)	0.05	0.19	0.06	1.59
WC	0.05 (−0.01; 0.11)	0.03	0.07	0.07	0.02 (−0.05; 0.09)	0.03	0.09	0.55	1.61
RAC	0.13 (0.01; 0.25)	0.06	0.10	**0.04**	0.11 (−0.08; 0.29)	0.09	0.12	0.26	2.55
NC	0.28 (0.11; 0.44)	0.08	0.25	**< 0.01**	0.33 (−0.01; 0.69)	0.17	0.19	0.06	4.11
BMI	0.11 (−0.02; 0.25)	0.06	0.05	0.09	0.03 (−0.16; 0.22)	0.09	0.08	0.74	1.97

ASF = abdominal skinfold; SSF = subscapular skinfold; TSF = triceps skinfold; CSF = calf skinfold; WC = waist circumference; RAC = relaxed arm circumference; NP = neck circumference; BMI = body mass index; β = regression coefficient; CI = confidence interval; SE = standard error; VIF = multicollinearity diagnosis;

* adjusted according to sex, bone age, CD4+ T lymphocytes, CD8+ T lymphocytes, viral load and physical activity.

For the females, direct associations were observed in simple analyses, such that ASF and TSF explained 20% and 18% of IR variability, respectively. In addition, direct associations with RAC (R² = 0.10) and NC (R² = 0.25) were observed. In the adjusted analyses, direct associations between IR and models with ASF (R² = 0.26) and TSF (R² = 0.31) were observed.

## DISCUSSION

The main results from the present study add to the current literature to show that higher values for peripheral and central skin folds are associated with IR.

SSF and CSF (males) and ASF and TSF (females) were directly associated with IR among these pediatric patients with HIV. Several studies have shown associations between different central and peripheral skinfolds and IR among HIV-infected children and adolescents,^25–28^ based on the assumption that accumulation of subcutaneous adiposity is associated with IR due to increased lipotoxicity.^29^ In this context, insulin favors entry of glucose into adipose tissue, which activates lipoprotein lipase, thus promoting storage of triglycerides and preventing the action of protein kinase, an intracellular enzyme that is capable of blocking insulin signaling pathways.^29^ However, in the context of HIV infection, the complexity of the scenario increases due to the adverse effects both of the virus itself and of ART. This is concomitant with possible increases in body fat and, consequently, the lipotoxic effect of lipodystrophy.^4^ Protease inhibitors (PIs) are believed to play an important role in the emergence of IR dyslipidemia and increased quantities of visceral adipose tissue.^1,4^

Specifically in relation to central skinfolds associated with IR, our data corroborate previous studies among children and adolescents of different ethnicities and without HIV diagnoses, regarding SSF^25,27^ and ASF.^30,31^ The android phenotype of body fat accumulation (more in the trunk) has been more associated with IR, which is explained by pancreatic β cell dysfunction due to formation of reactive oxygen species (ROS), which act on metabolic dysregulation to cause IR.^32^ Regarding peripheral skinfolds (CSF [male] and TSF [(female]), which demonstrated associations with IR, our data are consistent with the findings from a systematic review that demonstrated that peripheral subcutaneous fat was associated with IR.^33^

HIV-infected individuals undergoing ART treatment with protease inhibitors are predisposed to lipodystrophy syndrome (fat loss or accumulation) in peripheral regions such as arms and legs.^2^ Antiretroviral protease inhibitor treatment reduces the action of peroxisome proliferator-activated receptors (PPARy), thereby decreasing adiponectin levels and culminating in IR.^6^ Although there is no consensus on the association between different lipodystrophy phenotypes and IR in pediatric patients diagnosed with HIV, high insulin concentrations were found previously in children with lipohypertrophy, and less consistently in children with lipoatrophy.^4^

The skinfolds associated with IR differed according to sex (male/female). This may be explained by the existence of sexual dimorphism. In girls, as their age increases, estradiol hormone secretion also increases, which leads to fat accumulation in the arms and consequently increases the amount of adipocytes in the tricipital region.^24^ In boys, increasing secretion of testosterone hormone inhibits abdominal fat accumulation.^24^

Regarding the associations of anthropometric indicators with IR, the results from this study demonstrated the potential of skinfold analyses, such that associations were found with SSF and TSF among males, and with ASF and CSF among females. This is important from a practical point of view, for clinical use in monitoring the body composition and metabolic complications of HIV-infected children and adolescents, given that skinfold measurement is a low-cost alternative.

This study had some limitations, such as the fact that HOMA-IR was used as an indicator of glycemic homeostasis impairment. Nonetheless, this method is often used in clinical investigations. Other limitations related to the absence of any clinical diagnosis for lipodystrophy.

Among the strengths of this study, the analyses were controlled for potential confounders (sex, bone age, CD4+ T lymphocytes, CD8+ T lymphocytes, viral load and physical activity) in the multiple linear regression analyses, a strategy that had not previously been addressed in studies making correlations between anthropometric indicators and body fat among children and adolescents diagnosed with IR.

## CONCLUSIONS

In conclusion, SSF and CSF in males and ASF and TSF in females were directly associated with IR. It can be suggested that use of these anthropometric indicators should form part of the routine clinical follow-up for HIV-infected children and adolescents. These low-cost anthropometric measurements can contribute to risk stratification among children and adolescents with IR, and consequently may prevent metabolic complications such as type 2 diabetes and other cardiovascular consequences.
